# Gender differences in hypoxic acclimatization in cyclooxygenase‐2‐deficient mice

**DOI:** 10.14814/phy2.13148

**Published:** 2017-02-27

**Authors:** Kui Xu, Xiaoyan Sun, Girriso F. Benderro, Constantinos P. Tsipis, Joseph C. LaManna

**Affiliations:** ^1^ Department of Physiology and BiophysicsCase Western Reserve UniversityClevelandOhio

**Keywords:** Capillary density, hypoxia‐induced angiogenesis, hypoxic adaptation, prolonged hypoxia, sex differences

## Abstract

The aim of this study was to determine the effect of cyclooxygenase‐2 (COX‐2) gene deletion on the adaptive responses during prolonged moderate hypobaric hypoxia. Wild‐type (WT) and COX‐2 knockout (KO) mice of both genders (3 months old) were exposed to hypobaric hypoxia (~0.4 ATM) or normoxia for 21 days and brain capillary densities were determined. Hematocrit was measured at different time intervals; brain hypoxia‐inducible factor ‐1*α* (HIF‐1*α*), angiopoietin 2 (Ang‐2), brain erythropoietin (EPO), and kidney EPO were measured under normoxic and hypoxic conditions. There were no gender differences in hypoxic acclimatization in the WT mice and similar adaptive responses were observed in the female KO mice. However, the male KO mice exhibited progressive vulnerability to prolonged hypoxia. Compared to the WT and female KO mice, the male COX‐2 KO mice had significantly lower survival rate and decreased erythropoietic and polycythemic responses, diminished cerebral angiogenesis, decreased brain accumulation of HIF‐1*α*, and attenuated upregulation of VEGF, EPO, and Ang‐2 during hypoxia. Our data suggest that there are physiologically important gender differences in hypoxic acclimatization in COX‐2‐deficient mice. The COX‐2 signaling pathway appears to be required for acclimatization in oxygen‐limiting environments only in males, whereas female COX‐2‐deficient mice may be able to access COX‐2‐independent mechanisms to achieve hypoxic acclimatization.

## Introduction

The mammalian brain is dependent on timely availability of both oxygen and glucose for normal physiologic function. When mammals are exposed to chronic hypoxia, systemic and central adaptational changes allow them to acclimatize to the low oxygen environment (LaManna et al. 1992). The major long‐lasting systemic responses include hyperventilation, loss of body weight, and polycythemia through upregulated erythropoiesis. In the CNS, the major response is increased capillary density by angiogenesis over a 3‐week period of sustained exposure. Cyclooxygenase‐2 (COX‐2), an important marker of inflammation, is constitutively expressed in neurons, astrocytes, and endothelial cells in the brain under normal physiologic conditions (Kaufmann et al. 1996; Nogawa et al. 1997; Hirst et al. 1999) and upregulated by hypoxia (LaManna et al. 2004; Benderro and LaManna 2014). Enzymatic activity of COX‐2 in endothelial cells catalyzes the conversion of arachidonic acid to prostaglandin 2 (PGE2), which promotes angiopoietin‐2 (Ang‐2) expression near sites of vascular remodeling, inducing angiogenesis during hypoxia (Xu and LaManna 2006; Dore‐Duffy and LaManna 2007). The pathway of hypoxia‐inducible factor (HIF)‐mediated upregulation of vascular endothelial growth factor (VEGF) also involves in hypoxia‐induced angiogenesis (Wang and Semenza 1993; Levy et al. 1995). However, the interaction between these pathways remains unclear.

We recently reported the time course of HIF‐1*α*, VEGF, COX‐2, and Ang‐2 trended similarly during prolonged hypoxia (Benderro and LaManna 2014). It has been shown that the hypoxia‐induced COX‐2 activation may augment PGE2 release, resulting in increased accumulation of HIF‐1*α*, increased expression of VEGF, and enhanced angiogenesis (Casibang et al. 2001; Pai et al. 2001; Huang et al. 2005). Reduction of angiogenesis was observed in cornea of COX‐2 KO mice in an interleukin‐1*β*‐induced angiogenesis model, suggesting that the suppressed angiogenesis by inhibition of COX‐2 may be through the inhibition of HIF‐1*α* or VEGF expression (Liu et al. 1999; Jones et al. 2002). For example, NS ‐398, a COX‐2 selective inhibitor, suppressed hypoxia‐induced angiogenesis by reducing HIF‐1*α* or inhibition of HIF‐1 activity (Zhong et al. 2004) and markedly reduced hypoxia‐induced VEGF production (Liu et al. 1999), and this inhibitory effect could be reversed by exogenous PGE2 (Liu et al. 1999). Yanni et al. (2010) have demonstrated that hypoxia induces COX‐2, prostanoids production, and VEGF synthesis in retinal Müller cells, and that VEGF production is at least partially COX‐2‐dependent, suggesting that PGE2 mediates the VEGF response of Müller cells. However, some studies indicated that COX‐2 expression has no significant correlation with VEGF expression (Kim et al. 2011), and hypoxia‐driven HIF‐1*α* accumulation is independent of COX‐2 pathway (Stasinopoulos et al. 2009). On the other hand, though COX‐2 is mainly essential for induction of Ang‐2 during hypoxia (Pichiule et al. 2004; Yao et al. 2011) evidence has shown that VEGF also promotes Ang‐2 activities that favor vascular sprouting during hypoxia‐induced angiogenesis (Pichiule et al. 2004; LaManna et al. 2006).

The COX knockout mice have provided useful models for investigating the roles of the COX isoforms in normal physiology and various pathological states. Mice with genetic deletion of COX‐2 have been used to investigate the effects of COX‐2 deficiency on hypoxia‐induced vascular responses such as angiogenesis (Yanni et al. 2010). However, no study to date has examined sex differences in the COX‐2‐deficient mice in hypoxic acclimatization. Because there are examples of sex‐dependent differences in the COX‐2 KO mouse strain (Yang et al. 2005; Chillingworth et al. 2006; Robertson et al. 2006), it became apparent that it was necessary to study whether sex differences extended to the response to prolonged hypoxia. In this study, we investigated the role of COX‐2 on hypoxic acclimatization responses using COX‐2‐deficient mice of both genders in comparison with the wild‐type mice.

## Methods

### Animal preparation

The experimental protocol used in this study was approved by the Institutional Animal Care and Use Committee (IACUC) at Case Western Reserve University. COX‐2 heterozygous (±) males and females (B6; 129S7‐Ptgs2 ^tm 1 Jed^) were purchased from Jackson Laboratories (Bar Harbor, ME) and bred to produce both cyclooxygenase‐2 wild‐type (+/+, WT) and homozygous knockout (‐/‐, KO) mice from the same litter. Genotyping was performed using PCR analysis on DNA samples obtained from tail biopsies. All mice were housed and maintained at the Animal Resource Center on a 12:12‐h light/dark diurnal cycle with unrestricted access to food and water. Experiments were conducted in 3‐month‐old WT and COX‐2 KO mice of both genders.

### Chronic hypoxic exposure

As previously reported (Benderro and LaManna 2011, 2014), hypoxic mice (WT or KO) were kept in a hypobaric chamber in which a constant pressure of 300 mmHg (~0.4 atm, equivalent to 8% normobaric oxygen at sea level) was maintained. Pressure was periodically (maximum 1 h a day) returned to atmosphere for replenishment of food and water, cage cleaning, and body weight recording. Normoxic mice (littermates of WT or KO mice) were kept next to the chamber to ensure identical ambient conditions. For capillary density analysis, brains of mice were collected after 21 days of normoxic or hypoxic exposure. In a separate group of animals, blood and tissue samples were collected on 0, 1, 4, 7, 14, and 21 days of the exposure for the measurement of hematocrit and western blot analyses.

### Determination of cerebral capillary density

Brain microvascular density was determined by immunohistochemical staining for Glucose Transporter‐1 (GLUT‐1) and counting the number of GLUT‐1‐positive capillaries per unit area (N/mm^2^), as described previously (Benderro and LaManna 2011, 2014). Mice were deeply anesthetized with isoflurane and perfused transcardially with PBS (pH 7.4) and 4% paraformaldehyde. Brains were removed and immersed in 4% paraformaldehyde overnight at 4°C. The brain samples were dehydrated through graded alcohol and embedded in paraffin. Coronal serial sections (5 *μ*m) of frontal cortex (levels of Bregma 0.98 mm to 0.38 mm), (Paxinos and Franklin 2003) were made on a microtome. Sections were deparaffinized, rehydrated, and subjected to antigen retrieval at 90°C for 10 min in 0.1 mol/L sodium citrate buffer and incubated with 3% hydrogen peroxide. Slides were blocked with 10% normal horse serum for 1 h and then incubated with primary antibodies (anti‐Glut‐1, Santa Cruz, CA) at 4°C overnight. After three serial washes with 0.1 mol/L PBS‐tween solution, the secondary antibody (1:200, Vector Labs, Burlingame, CA) was applied. The slides were washed again and incubated in Vectastain ABC Elite reagent (Vector Labs, Burlingame, CA) for 30 min and then developed using diaminobenzidine. After dehydration and coverslipping, images were taken with a SPOT digital camera in conjunction with a Nikon E600 Eclipse microscope. Images spanning the entire depth of the parietal cortex were resolved at 200× optical resolution. Adobe Photoshop CS5 and ImageJ were used to count positively stained microvessels, less than 20 *μ*m in diameter, to determine the capillary density (number per mm^2^ of brain tissue). For each brain, at least four different GLUT‐1‐stained sections were averaged for quantification. Each quantified section was at least 50 *μ*m apart from the subsequent quantified section.

### Western blot analysis

Western blot analysis was performed on brain and kidney samples as described previously (Benderro and LaManna 2011). Mice were anesthetized with isoflurane and decapitated. Brain and kidney samples were dissected and stored at −80°C. Tissue samples were homogenized using ice‐cold lysis buffer (50 mmol/L Tris‐HCl, pH 8.0; 150 mmol/L NaCl, 1% Nonidet P‐40, 0.5% sodium deoxycholate, and 0.1% SDS) supplemented with protease inhibitors (Complete; Roche, Indianapolis, IN). Whole cell lysates (75 *μ*g of protein) were electrophoresed on an SDS‐PAGE 30% acrylamide gel and transferred to a nitro‐cellulose membrane. Membranes were blocked in 10% nonfat dry milk blocking buffer and incubated with specific primary antibodies, respectively, anti‐EPO (1:500, Santa Cruz), anti‐HIF‐1*α* (1:500, R&D system), anti‐VEGF‐A (1:1000; Santa Cruz), Ang‐2 (1:200; Millipore Co., Billerica, MA), anti‐*β*‐Actin (1: 2000, Santa Cruz), and anti‐*β*‐tubulin (1:2000, Cell Signaling). The membranes were washed with TBS‐tween washing buffer followed by incubating with corresponding horseradish peroxidase‐conjugated secondary antibodies. After three washes with TBS‐tween, immunoreactive protein bands were visualized using enhanced chemiluminescence detection system (Thermo Scientific) and subsequent exposure to Hyperfilm (Thermo Scientific). SigmaScan Pro was used to quantify densitometry of protein bands and normalized to *β*‐tubulin or *β*‐actin.

### Statistical analysis

All values were presented as mean ± SD. Statistical analyses were performed using SPSS v 20.0 for Windows. Group comparisons were made by one‐way analysis of variance (ANOVA) using Tukey's statistic. The comparison between any two groups was analyzed with a t‐test for paired sample, two‐tailed. The survival analysis was performed using a Wilcoxon (Gehan) survival analysis. Significance was considered at the level of *P* < 0.05.

## Results

### Overall survival during hypoxic exposure

**Figure 1:**
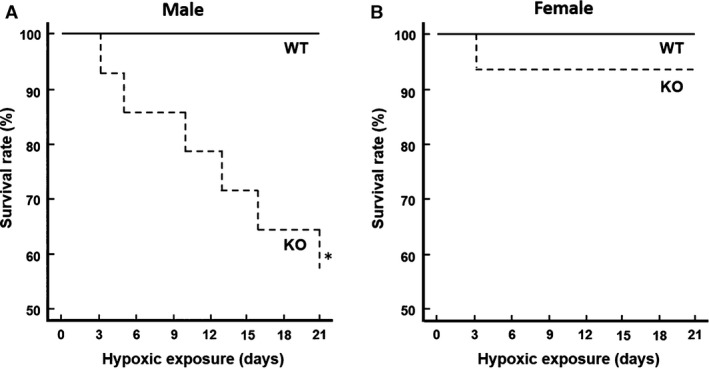
Overall survival in the wild‐type (WT) and the COX‐2 knockout (KO) mice during 21‐day hypoxic exposure. (A) In males, the KO group had a significantly lower survival rate compared to the WT group (KO: 57%, 8/14; WT: 100%, 21/21). (B) In females, the survival rate was similar in the WT and the KO groups (WT: 100%, 21/21; KO: 94%, 15/16). * indicates significant difference from the WT group, Wilcoxon survival analysis, *P* < 0.05.

The overall survival was monitored in mice exposed to hypoxia for 21 days (Fig. 1). All WT mice (male and female, *n* = 21 each) successfully survived the whole length of 21‐day hypoxic exposure. However, the male KO mice had continuous death between 3 and 20 days of exposure and only about half survived (58%, 8/14) for 21 days. On the contrary, the female KO mice had similar survival profile compared to the WT mice, all but one female KO mouse (15/16) survived the full hypoxic exposure.

### Body weight change during hypoxia

**Figure 2:**
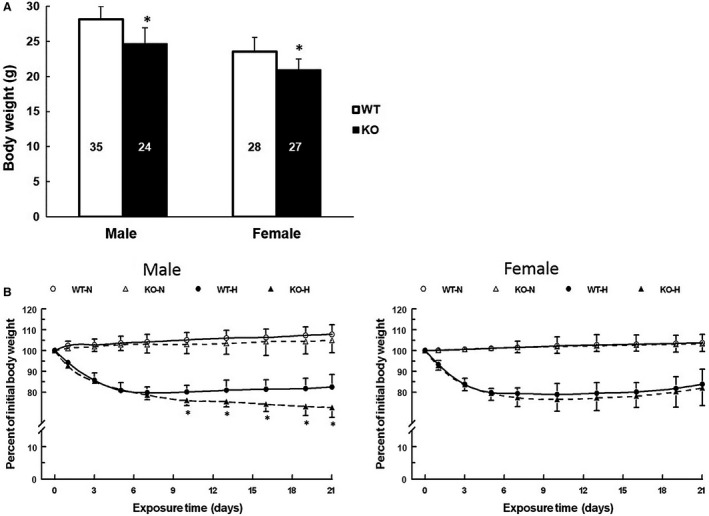
(A) Body weights of age‐matched wild‐type (WT) and COX‐2 knockout (KO) mice before hypoxic or normoxic exposure. Values are mean ± SD, * indicates significant difference (t‐test, *P* < 0.05) from the WT group with same sex; n for each group is indicated on each bar. (B) Change in body weight (% of initial weight) in mice during normoxic or hypoxic exposure. WT‐N: WT normoxic, *n* = 13 each for males and females; KO‐N: KO normoxic, *n* = 10 each for males and females; WT‐H: WT hypoxic, *n* = 21 each for males and females; KO‐H: KO hypoxic, *n* = 8 and 15 for males and females, respectively. Values are mean ± SD, * indicates significant difference (t‐test, *P* < 0.05) from the WT group at the same time point with same exposure condition.

As seen in Figure 2, baseline body weights and the change in body weights during 21‐day normoxic or hypoxic exposure were measured in the age‐matched WT (male: *n* = 39; female: *n* = 28) and KO mice (male: *n* = 24; female: *n* = 27). Both male and female KO mice had significantly lower body weights compared to their corresponding WT mice (grams, male: 25 ± 2 vs. 28 ± 1.8, female: 21 ± 1.6 vs. 24 ± 2.2, Fig. 2A). In the normoxic groups, the body weight profiles of WT and KO groups were similar in both male and female mice (Fig. 2B). During the first week of hypoxia, all groups had a significant and similar magnitude of body weight loss (about 20%) compared to their corresponding pre‐hypoxic baselines. After day 7, the WT mice (both male and female) and female KO mice started to regain body weight gradually and reached about 83% of normoxic baseline at day 21. However, the surviving male KO mice had continuous body weight loss during the entire length of hypoxic exposure; the body weight was only about 73% of normoxic baseline at 21 days of hypoxia (Fig. 2B).

### Hematocrit change during hypoxia

**Figure 3:**
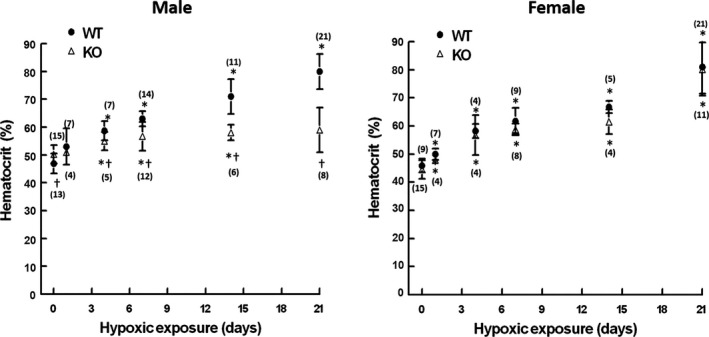
Change in hematocrit in the wild‐type (WT) and the COX‐2 knockout (KO) mice during hypoxic exposure. Values are mean ± SD. n of each group is indicated for each time point, normoxic, 1, 4, 7, 14, and 21 days of hypoxia. * indicates significant difference (t‐test, *P* < 0.05) from the corresponding normoxic baseline; † indicates significant difference (t‐test, *P* < 0.05) from the WT group at the same time point.

The time course of hematocrit change during hypoxia was measured in WT and KO mice (Fig. 3). In the male mice, the KO group had a slightly higher normoxic hematocrit compared to the WT group (%, 50 ± 3, *n* = 13 vs. 47 ± 4, *n* = 15, *P* < 0.05). During hypoxia, the hematocrit in the male and female WT mice increased gradually and reached about 60% at day 4, and the hematocrit was about 80% at day 21. The hypoxia induced similar change in hematocrit in the female WT and KO groups compared to the WT mice. However, the hematocrit in the male KO group only reached 55% at 4 days of hypoxia and was sustained at that level throughout the remaining of exposure. In females, the WT and the KO mice had similar baseline hematocrit. The WT and KO groups had similar trend of hematocrit change during hypoxia; the hematocrit was reached about 80% at day 21 of exposure (Fig. 3).

### EPO expression in kidney

**Figure 4:**
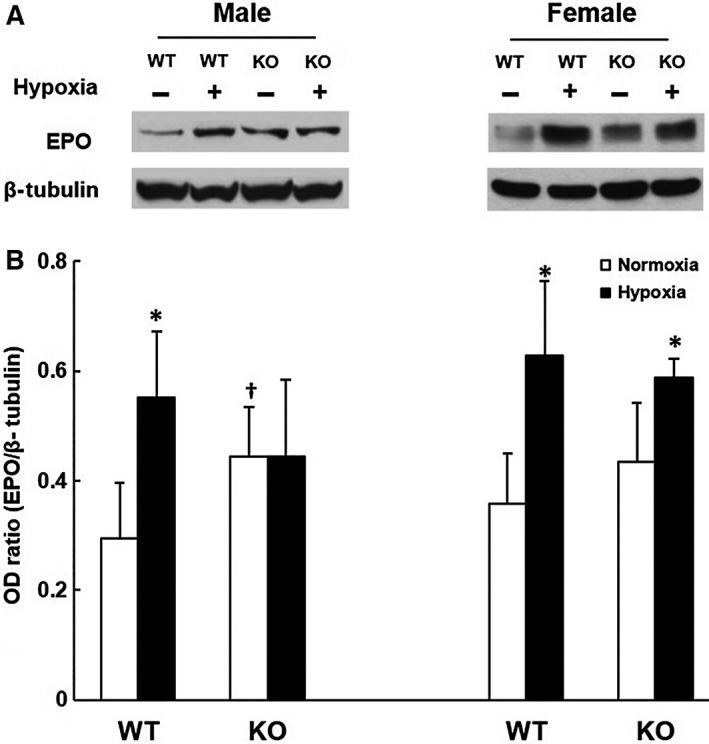
EPO expression in kidney in the wild‐type (WT) and the COX‐2 knockout (KO) mice under normoxia and 7‐day hypoxia. (A) Representative Western blot analysis of normoxic control and 7‐day hypoxia in male and female mice, respectively. (B) Optical density ratios of EPO normalized to *β*‐tubulin. Values are mean ± SD,* n* = 6 for each group. * indicates significant difference (t‐test, *P* < 0.05) from the corresponding normoxic baseline; † indicates significant difference (t‐test, *P* < 0.05) from the WT group with same exposure condition.

The kidney EPO protein level was measured in the WT and the KO mice under normoxic and 7‐day hypoxic conditions (Fig. 4). The 7‐day time point was chosen because we have previously reported that the peak elevation of EPO occurred at 7 days of hypoxia in kidney during chronic hypoxia in mice (Benderro and LaManna 2013). In the male mice, the normoxic baseline EPO in the KO group was significantly higher (50% increase) than the WT mice. The kidney EPO increased over onefold at 7‐day hypoxia in the WT mice but remained unchanged in the KO mice. In females, the WT and the KO mice had a similar EPO baseline, and the EPO level was significantly increased in both WT and KO groups at 7‐day hypoxia.

### Microvascular density in cerebral cortex

**Figure 5:**
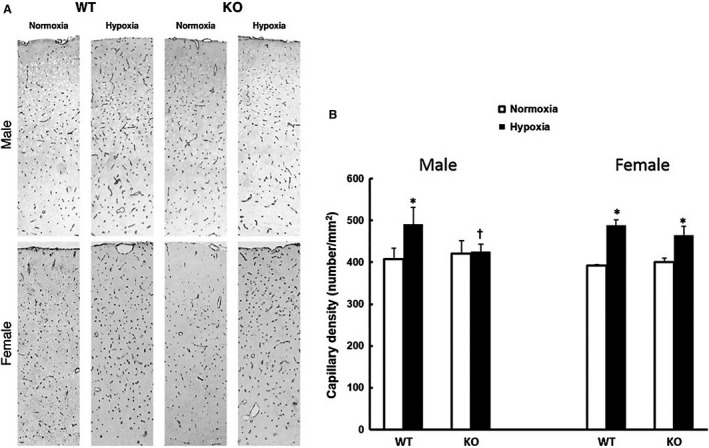
Microvascular density in cerebral cortex in the wild‐type (WT) and the COX‐2 knockout (KO) mice under normoxia and 21‐day hypoxia. (A) Representative images of GLUT‐1 immunohistostaining from WT and KO mice of both genders. (B) Capillary density (Number/mm^2^) as identified by the GLUT‐1 positive staining in brain cortex. Values are mean ± SD,* n* = 6 for each group. * indicates significant difference (t‐test, *P* < 0.05) from the corresponding normoxic baseline; † indicates significant difference (t‐test, *P* < 0.05) from the WT group at the same exposure condition.

Cerebral capillary density (N/mm^2^) was identified by GLUT‐1 immunostaining and quantified as described previously (Benderro and LaManna 2011). As seen in Figure 5, the baseline capillary density (N/mm^2^) was similar in all groups, WT or KO, male or female (male, WT: 408 ± 20, KO: 410 ± 22; Female, WT: 392 ± 3, KO: 400 ± 10, *n* = 6 each). There were no sex differences in normoxic capillary density in the WT or in the KO mice. Consistent with previous studies (Benderro and LaManna 2011), capillary density in the male WT mice increased about 20% after 3 weeks of hypoxia (491 ± 45, *n* = 6). However, the capillary density in the male KO mice remained unchanged (426 ± 18, *n* = 6) after hypoxic exposure. In the female mice, both WT and KO had significantly higher capillary densities (490 ± 13 and 465 ± 21, respectively, *n* = 6 each) compared to their corresponding normoxic baselines.

### Expression of HIF‐1*α*, VEGF, EPO, and Ang‐2 in cerebral cortex

**Figure 6:**
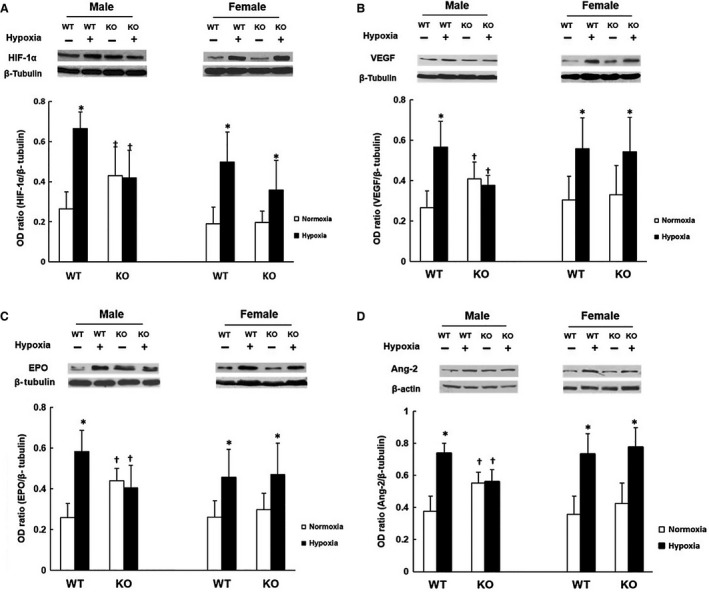
Western blot analysis of HIF‐1*α*, VEGF, EPO, and ANG‐2 in cerebral cortex in the wild‐type (WT) and the COX‐2 knockout (KO) mice under normoxia and 7‐day hypoxia. (A) HIF‐1*α*. (B) VEGF. (C) EPO. (D) Ang‐2. For A to D, upper panel: Representative western blot analysis of normoxic control and 7‐day hypoxia in male and female mice, respectively. Lower panel: Optical density ratios of respective protein normalized to *β*‐tubulin or *β*‐actin. Values are mean ± SD,* n* = 4–8 for each group. * indicates significant difference (t‐test, *P* < 0.05) from the corresponding normoxic baseline; † indicates significant difference (t‐test, *P* < 0.05) from the WT group at the same exposure condition.

The western blot analysis of HIF‐1*α*, EPO, VEGF, and Ang‐2 were performed in the cerebral cortex of WT and KO mice under normoxic and hypoxic conditions (Fig. 6). In the male mice, the KO group had a significantly higher (~50% increase) normoxic baseline HIF‐1*α*, EPO, VEGF, and Ang‐2 compared to the WT group. At 7 days of hypoxia, levels of HIF‐1*α*, EPO, VEGF, and Ang‐2 were significantly increased by twofold to 2.5‐folds in the WT group, but the male WT mice exhibited no hypoxia‐induced upregulation of these proteins. However, in the females, the WT and the KO mice had similar normoxic baselines of HIF‐1*α*, EPO, VEGF, and Ang‐2. At 7 days of hypoxia, the levels of the above proteins were increased significantly in both WT and KO groups, and by a similar magnitude.

## Discussion

In this study, we investigated the role of COX‐2 on acclimatization to prolonged moderate hypoxia using COX‐2‐deficient mice of both genders. We found that there were no gender differences in hypoxic acclimatization in the WT mice; however, remarkable gender differences were observed in the COX‐2‐deficient mice. The male KO mice exhibited progressive vulnerability to prolonged hypoxia, as demonstrated by decreased survival, diminished erythropoietic response, and lack of hypoxia‐induced cerebral capillary angiogenesis during hypoxic exposure. Unexpectedly, female KO mice demonstrated no deficiency in adaptive responses compared to the WT mice.

The male KO mice had continuous body weight loss and death during the entire length of hypoxic exposure, suggesting that there was no critical survival window for the male KO mice. The progressive mortality during hypoxia in the male KO mice may be also related to the diminished erythropoietic and polycythemic responses. The hematologic acclimatization response, driven by kidney‐produced erythropoietin, enables the maintenance of oxygen content in blood and improvement of tissue oxygenation despite decreased arterial partial pressure of O_2_ (PaO_2_) during hypoxia (Xu et al. 2004). We have previously reported that kidney EPO was elevated throughout the 21‐day hypoxic period and peaked between 7 and 14 days (Benderro and LaManna 2013); hematocrit increased with continued hypoxia, doubling by 21 days (Benderro and LaManna 2011, 2013). The relatively elevated basal kidney EPO and hematocrit level in the male KO mice may indicate a hypoxia‐like state in the kidney tissue and a diminishing of further hypoxic sensitivity, due to the deficiency of COX‐2.

Cerebral vascular remodeling through angiogenesis is the major CNS acclimatization response to prolonged hypoxia (LaManna et al. 2004). Reduction in angiogenesis was observed in cornea of COX‐2 KO mice in an interleukin‐1*β*‐induced angiogenesis model (Kuwano et al. 2004). It has been shown in vitro that hypoxia‐induced VEGF production was diminished in COX‐2 KO mouse retinal Müller cells (Yanni et al. 2010). In our study, we observed that the absence of COX‐2 in males resulted in attenuated HIF‐1*α* accumulation, response deficits in downstream gene products EPO and VEGF during hypoxia, the suppressed Ang‐2 upregulation and the overall failure to induce new capillary formation in the cerebral cortex, suggesting that HIF‐1*α*/VEGF pathway can be regulated by COX‐2 but the effect appears to be gender‐dependent. The relatively higher baseline of HIF‐1*α*, VEGF, EPO, and Ang‐2 in the male KO mice may reflect the hypoxia‐like state in these mice, as the elevated EPO baseline level we observed in kidney tissue. In addition, the attenuated HIF‐1*α* upregulation may be also responsible for the progressive vulnerability to prolonged hypoxia in the male KO mice. HIF‐1*α* is a nuclear factor associated with neuroprotection via regulation of energy metabolism and is a key regulator of oxygen homeostasis during hypoxia (Semenza 1999). HIF‐1*α* regulates genes related to glucose metabolism, angiogenesis, and erythropoiesis to promote cell survival (Bergeron et al. 2000; Semenza 2000; Kiriakidis et al. 2007).

The gender differences caused by COX‐2 deletion or inhibition have been observed in other studies. The elevated level of estrogen is positively associated with cerebral blood flow (Kastrup et al. 1999) and is favorable on recovery following stroke (McCullough et al. 2001; Manwani et al. 2015). A recent human study has indicated that in females, hypoxia‐mediated cerebral vasodilation is similar across early and late follicular phases and is not affected by COX inhibition (Peltonen et al. 2016). It has been shown that the male 129/COX‐2 ‐/‐ mice exhibit malignant hypertension, overt proteinuria, and severe renal abnormalities compared to milder defects in the female mice (Yang et al. 2005). In a model of arthritis and inflammatory pain, both disease severity and nociception, COX‐2 knockout females exhibited reduced edema and joint destruction compared with male knockouts or wild types of either sex (Chillingworth et al. 2006). Genetic deletion of COX‐2 may also have a sex‐dependent effect on maintenance of normal bone microarchitecture and density in mice. It has been shown that in 4‐month‐old COX‐2 knockout mice, the females had normal bone geometry and trabecular microarchitecture, whereas the age‐matched males exhibited reduced bone volume fraction within the distal femoral metaphysis (Robertson et al. 2006). In humans, nonsteroidal anti‐inflammatory drugs (NSAIDs), which have been linked to their ability to inhibit inducible COX‐2 at sites of inflammation, may produce different responses in men and women; for example, ibuprofen has little effect on noninflammatory experimental pain in women, but is effective in men (Chillingworth et al. 2006). Aspirin, a NSAID simultaneously inhibits COX‐1 and COX‐2 isoforms (Warner and Mitchell 2004), has been shown to impair the wound healing process in female, but not male mice. It also showed that the expression of von Willebrand factor (vWF, an endothelial cell marker) and VEGF was the same in the female and male control groups, but was higher in the female aspirin‐treated group compared with the male aspirin‐treated group (dos Santos and Monte‐Alto‐Costa 2013). It has been reported that estrogen stimulates angiogenesis by a direct effect on endothelial cells during wound healing (Gilliver et al. 2008). Sex‐dependent effect of COX‐2 inhibition was also observed in cognitive performance in mouse, suggesting that COX‐2 activity may influence mnemonic processes in a sex‐dependent manner (Guzman et al. 2009). These findings suggest the importance of studying subjects of both genders in rodent models of neurodegenerative disorders and developing of treatment strategies selectively according to gender.

In conclusion, we found that there were no gender differences in hypoxic acclimatization in the WT mice. While female COX‐2‐deficient mice successfully responded to hypoxic exposure in a manner similar to the WT mice, the male COX‐2‐deficient mice were incapable of physiological acclimatization. Our data suggest that there are physiologically important gender differences in hypoxic acclimatization in COX‐2‐deficient mice. The COX‐2 signaling pathway appears to be required for successful hypoxic acclimatization in males, however, female COX‐2‐deficient mice may acclimatize to hypoxia through COX‐2‐independent mechanisms.

## Conflict of Interest

None declared.

## References

[phy213148-bib-0001] Benderro, G. F. , and J. C. LaManna . 2011 Hypoxia‐induced angiogenesis is delayed in aging mouse brain. Brain Res. 1389:50–60. 21402058 10.1016/j.brainres.2011.03.016 PMC3082052

[phy213148-bib-0002] Benderro, G. F. , and J. C. LaManna . 2013 Kidney EPO expression during chronic hypoxia in aged mice. Adv. Exp. Med. Biol. 765:9–14. 22879008 10.1007/978-1-4614-4989-8_2

[phy213148-bib-0003] Benderro, G. F. , and J. C. LaManna . 2014 HIF‐1alpha/COX‐2 expression and mouse brain capillary remodeling during prolonged moderate hypoxia and subsequent re‐oxygenation. Brain Res. 1569:41–47. 24796880 10.1016/j.brainres.2014.04.035 PMC4066660

[phy213148-bib-0004] Bergeron, M. , J. M. Gidday , A. Y. Yu , G. L. Semenza , D. M. Ferriero , and F. R. Sharp . 2000 Role of hypoxia‐inducible factor‐1 in hypoxia‐induced ischemic tolerance in neonatal rat brain. ‎*Ann* . Neurol. 48:285–296. 10976634

[phy213148-bib-0005] Casibang, M. , S. Purdom , S. Jakowlew , L. Neckers , F. Zia , P. Ben‐Av , et al. 2001 Prostaglandin E2 and vasoactive intestinal peptide increase vascular endothelial cell growth factor mRNAs in lung cancer cells. Lung Cancer 31:203–212. 11165399 10.1016/s0169-5002(00)00168-9

[phy213148-bib-0006] Chillingworth, N. L. , S. G. Morham , and L. F. Donaldson . 2006 Sex differences in inflammation and inflammatory pain in cyclooxygenase‐deficient mice. Am. J. Physiol. Regul. Integr. Comp. Physiol. 291:R327–R334. 16556900 10.1152/ajpregu.00901.2005

[phy213148-bib-0007] Dore‐Duffy, P. , and J. C. LaManna . 2007 Physiologic angiodynamics in the brain. *Antioxid. Redox* . Signal 9:1363–1371. 10.1089/ars.2007.1713 17627476

[phy213148-bib-0008] Gilliver, S. C. , J. P. Ruckshanthi , M. J. Hardman , T. Nakayama , and G. S. Ashcroft . 2008 Sex dimorphism in wound healing: the roles of sex steroids and macrophage migration inhibitory factor. Endocrinology 149:5747–5757. 18653719 10.1210/en.2008-0355

[phy213148-bib-0009] Guzman, C. B. , K. A. Graham , L. A. Grace , and A. H. Moore . 2009 Sex‐dependent effect of cyclooxygenase‐2 inhibition on mouse spatial memory. Behav. Brain Res. 199:355–359. 19162088 10.1016/j.bbr.2009.01.005 PMC2693915

[phy213148-bib-0010] Hirst, W. D. , K. A. Young , R. Newton , V. C. Allport , D. R. Marriott , and G. P. Wilkin . 1999 Expression of COX‐2 by normal and reactive astrocytes in the adult rat central nervous system. Mol. Cell Neurosci. 13:57–68. 10049531 10.1006/mcne.1998.0731

[phy213148-bib-0011] Huang, S. P. , M. S. Wu , C. T. Shun , H. P. Wang , C. Y. Hsieh , M. L. Kuo , et al. 2005 Cyclooxygenase‐2 increases hypoxia‐inducible factor‐1 and vascular endothelial growth factor to promote angiogenesis in gastric carcinoma. J. Biomed. Sci. 12:229–241. 15864753 10.1007/s11373-004-8177-5

[phy213148-bib-0012] Jones, M. K. , I. L. Szabo , H. Kawanaka , S. S. Husain , and A. S. Tarnawski . 2002 von Hippel Lindau tumor suppressor and HIF‐1alpha: new targets of NSAIDs inhibition of hypoxia‐induced angiogenesis. FASEB J. 16:264–266. 11772947 10.1096/fj.01-0589fje

[phy213148-bib-0013] Kastrup, A. , V. Happe , C. Hartmann , and M. Schabet . 1999 Gender‐related effects of indomethacin on cerebrovascular CO2 reactivity. J. Neurol. Sci. 162:127–132. 10202978 10.1016/s0022-510x(98)00288-3

[phy213148-bib-0014] Kaufmann, W. E. , P. F. Worley , J. Pegg , M. Bremer , and P. Isakson . 1996 COX‐2, a synaptically induced enzyme, is expressed by excitatory neurons at postsynaptic sites in rat cerebral cortex. Proc. Natl Acad. Sci. 93:2317–2321. 8637870 10.1073/pnas.93.6.2317 PMC39793

[phy213148-bib-0015] Kim, A. R. , J. Y. Lim , D. C. Jeong , G. Park , B. C. Lee , and C. K. Min . 2011 Blockade of vascular endothelial growth factor (VEGF) aggravates the severity of acute graft‐versus‐host disease (GVHD) after experimental allogeneic hematopoietic stem cell transplantation (allo‐HSCT). Immune Netw. 11:368–375. 22346777 10.4110/in.2011.11.6.368 PMC3275706

[phy213148-bib-0016] Kiriakidis, S. , M. A. Esteban , and P. H. Maxwell . 2007 Genetic insights into the hypoxia‐inducible factor (HIF) pathway. Adv. Enzyme Regul. 47:288–306. 17335877 10.1016/j.advenzreg.2006.12.009

[phy213148-bib-0017] Kuwano, T. , S. Nakao , H. Yamamoto , M. Tsuneyoshi , T. Yamamoto , M. Kuwano , et al. 2004 Cyclooxygenase 2 is a key enzyme for inflammatory cytokine‐induced angiogenesis. FASEB J. 18:300–310. 14769824 10.1096/fj.03-0473com

[phy213148-bib-0018] LaManna, J. C. , L. M. Vendel , and R. M. Farrell . 1992 Brain adaptation to chronic hypobaric hypoxia in rats. J. Appl. Physiol. 72:2238–2243. 1629078 10.1152/jappl.1992.72.6.2238

[phy213148-bib-0019] LaManna, J. C. , J. C. Chavez , and P. Pichiule . 2004 Structural and functional adaptation to hypoxia in the rat brain. J. Exp. Biol. 207:3163–3169. 15299038 10.1242/jeb.00976

[phy213148-bib-0020] LaManna, J. C. , X. Sun , A. D. Ivy , and N. L. Ward . 2006 Is cycloxygenase‐2 (COX‐2) a major component of the mechanism responsible for microvascular remodeling in the brain? Adv. Exp. Med. Biol. 578:297–303. 16927708 10.1007/0-387-29540-2_47

[phy213148-bib-0021] Levy, A. P. , N. S. Levy , S. Wegner , and M. A. Goldberg . 1995 Transcriptional regulation of the rat vascular endothelial growth factor gene by hypoxia. J. Biol. Chem. 270:13333–13340. 7768934 10.1074/jbc.270.22.13333

[phy213148-bib-0022] Liu, X. H. , A. Kirschenbaum , S. Yao , M. E. Stearns , J. F. Holland , K. Claffey , et al. 1999 Upregulation of vascular endothelial growth factor by cobalt chloride‐simulated hypoxia is mediated by persistent induction of cyclooxygenase‐2 in a metastatic human prostate cancer cell line. *Clin* . Exp. Metastasis 17:687–694. 10.1023/a:1006728119549 10919714

[phy213148-bib-0023] Manwani, B. , K. Bentivegna , S. E. Benashski , V. R. Venna , Y. Xu , A. P. Arnold , et al. 2015 Sex differences in ischemic stroke sensitivity are influenced by gonadal hormones, not by sex chromosome complement. J. Cereb. Blood Flow Metab. 35:221–229. 25388681 10.1038/jcbfm.2014.186 PMC4426738

[phy213148-bib-0024] McCullough, L. D. , N. J. Alkayed , R. J. Traystman , M. J. Williams , and P. D. Hurn . 2001 Postischemic estrogen reduces hypoperfusion and secondary ischemia after experimental stroke. Stroke 32:796–802. 11239204 10.1161/01.str.32.3.796

[phy213148-bib-0025] Nogawa, S. , F. Zhang , M. E. Ross , and C. Iadecola . 1997 Cyclo‐oxygenase‐2 gene expression in neurons contributes to ischemic brain damage. J. Neurosci. 17:2746–2755. 9092596 10.1523/JNEUROSCI.17-08-02746.1997 PMC6573095

[phy213148-bib-0026] Pai, R. , I. L. Szabo , B. A. Soreghan , S. Atay , H. Kawanaka , and A. S. Tarnawski . 2001 PGE(2) stimulates VEGF expression in endothelial cells via ERK2/JNK1 signaling pathways. Biochem. Biophys. Res. Commun. 286:923–928. 11527387 10.1006/bbrc.2001.5494

[phy213148-bib-0027] Paxinos, G. , and K. B. J. Franklin . 2003 The Mouse brain in stereotaxic coordinates. Academic Press, San Diego, CA.

[phy213148-bib-0028] Peltonen, G. L. , J. W. Harrell , B. P. Aleckson , K. M. LaPlante , M. K. Crain , and W. G. Schrage . 2016 Cerebral blood flow regulation in women across menstrual phase: differential contribution of cyclooxygenase to basal, hypoxic, and hypercapnic vascular tone. Am. J. Physiol. Regul. Integr. Comp. Physiol. 311:R222–R231. 27225949 10.1152/ajpregu.00106.2016 PMC5008661

[phy213148-bib-0029] Pichiule, P. , J. C. Chavez , and J. C. LaManna . 2004 Hypoxic regulation of angiopoietin‐2 expression in endothelial cells. J. Biol. Chem. 279:12171–12180. 14702352 10.1074/jbc.M305146200

[phy213148-bib-0030] Robertson, G. , C. Xie , D. Chen , H. Awad , E. M. Schwarz , R. J. O'Keefe , et al. 2006 Alteration of femoral bone morphology and density in COX‐2‐/‐ mice. Bone 39:767–772. 16731065 10.1016/j.bone.2006.04.006 PMC2647994

[phy213148-bib-0031] dos Santos, J. S. , and A. Monte‐Alto‐Costa . 2013 Female, but not male, mice show delayed cutaneous wound healing following aspirin administration. Clin. Exp. Pharmacol. Physiol. 40:90–96. 23240590 10.1111/1440-1681.12043

[phy213148-bib-0032] Semenza, G. L. 1999 Regulation of mammalian O2 homeostasis by hypoxia‐inducible factor 1. Annu. Rev. Cell Dev. Biol. 15:551–578. 10611972 10.1146/annurev.cellbio.15.1.551

[phy213148-bib-0033] Semenza, G. L. 2000 HIF‐1: mediator of physiological and pathophysiological responses to hypoxia. J. Appl. Physiol. 88:1474–1480. 10749844 10.1152/jappl.2000.88.4.1474

[phy213148-bib-0034] Stasinopoulos, I. , D. R. O'Brien , and Z. M. Bhujwalla . 2009 Inflammation, but not hypoxia, mediated HIF‐1alpha activation depends on COX‐2. Cancer Biol. Ther. 8:31–35. 19390242 10.4161/cbt.8.1.7079 PMC3058789

[phy213148-bib-0035] Wang, G. L. , and G. L. Semenza . 1993 General involvement of hypoxia‐inducible factor 1 in transcriptional response to hypoxia. Proc. Natl Acad. Sci. 90:4304–4308. 8387214 10.1073/pnas.90.9.4304 PMC46495

[phy213148-bib-0036] Warner, T. D. , and J. A. Mitchell . 2004 Cyclooxygenases: new forms, new inhibitors, and lessons from the clinic. FASEB J. 18:790–804. 15117884 10.1096/fj.03-0645rev

[phy213148-bib-0037] Xu, K. , and J. C. LaManna . 2006 Chronic hypoxia and the cerebral circulation. J. Appl. Physiol. 100:725–730. 16421279 10.1152/japplphysiol.00940.2005

[phy213148-bib-0038] Xu, K. , M. A. Puchowicz , and J. C. LaManna . 2004 Renormalization of regional brain blood flow during prolonged mild hypoxic exposure in rats. Brain Res. 1027:188–191. 15494170 10.1016/j.brainres.2004.08.046

[phy213148-bib-0039] Yang, T. , Y. G. Huang , W. Ye , P. Hansen , J. B. Schnermann , and J. P. Briggs . 2005 Influence of genetic background and gender on hypertension and renal failure in COX‐2‐deficient mice. Am. J. Physiol. Renal. Physiol. 288:F1125–F1132. 15613621 10.1152/ajprenal.00219.2004

[phy213148-bib-0040] Yanni, S. E. , G. W. McCollum , and J. S. Penn . 2010 Genetic deletion of COX‐2 diminishes VEGF production in mouse retinal Muller cells. Exp. Eye Res. 91:34–41. 20398651 10.1016/j.exer.2010.03.019 PMC2879458

[phy213148-bib-0041] Yao, L. , F. Liu , L. Hong , L. Sun , S. Liang , K. Wu , et al. 2011 The function and mechanism of COX‐2 in angiogenesis of gastric cancer cells. J. Exp. Clin. Cancer Res. 30:13. 21266034 10.1186/1756-9966-30-13 PMC3039621

[phy213148-bib-0042] Zhong, H. , M. Willard , and J. Simons . 2004 NS398 reduces hypoxia‐inducible factor (HIF)‐1alpha and HIF‐1 activity: multiple‐level effects involving cyclooxygenase‐2 dependent and independent mechanisms. Int. J. Cancer 112:585–595. 15382039 10.1002/ijc.20438

